# Using Coding to Improve Executive Functioning in Children with Sickle Cell Disease: A Multiple-Baseline Single-Case Study

**DOI:** 10.3390/jintelligence14040055

**Published:** 2026-04-01

**Authors:** Barbara Arfé, Maria Elisa delle Fave, Chiara Montuori, Lucia Ronconi, Sofia Carbone, Raffaella Colombatti

**Affiliations:** 1 Department of Developmental Psychology and Socialization, University of Padova, 35131 Padova, Italy; chiara.montuori@unipd.it (C.M.); sofia.carbone@studenti.unipd.it (S.C.); 2 Human Inspired Technologies (HIT) Research Center, University of Padova, 35131 Padova, Italy; 3 Department of Women’s and Child’s Health, Pediatric Hematology-Oncology Unit, Azienda Ospedaliera-University of Padova, 35122 Padova, Italy; medellefave@gmail.com (M.E.d.F.); raffaella.colombatti@unipd.it (R.C.); 4 School of Psychology, University of Padova, 35131 Padova, Italy; l.ronconi@unipd.it

**Keywords:** coding, computational thinking, cognitive training, developmental disabilities, executive functions, sickle-cell disease

## Abstract

Executive function (EF) impairments are common in children with intellectual and developmental disabilities and have a significant impact on learning and daily life. Cognitive training programs aimed at strengthening EFs may show limited feasibility and generalization. However, recent studies suggest that ecological, curriculum-embedded problem-solving activities may be more promising. This multiple-baseline single-case study tested the feasibility and efficacy of a short computational thinking and coding intervention based on problem-solving for children with sickle cell disease, a hemoglobinopathy associated with cognitive decline and EF deficits. The trial followed the What Works Clearinghouse (WWC) Version 5 guidelines for single-case research. Three 7–8-year-old children with lower-range IQ (71–82) and EF impairments completed 11 coding sessions over 5–6 weeks using *code.org*, with pre/post assessments of non-verbal EF (planning, inhibition, and switching), and verbal EF skills (verbal working memory, phonological fluency and semantic fluency). Results showed 100% adherence to the intervention, significant improvement in coding (IRD range = 0.69–0.79), with positive transfer effects on nonverbal planning skills (gains > 2 z-scores) and also verbal fluency (z-score gains ranging from 0.47 to 1.04). Inter-individual variability in effects was related to the child’s individual cognitive profile. Findings suggest that problem-solving, coding-based activities can be feasible and potentially beneficial for children with significant EF impairments.

## 1. Introduction

Over the past decade, research in developmental disabilities and special education has increasingly focused on identifying interventions that effectively promote executive function (EF) development (Bombonato et al., 2024; Takacs & Kassai, 2019). Meta-analyses indicate that, in children with and without developmental disabilities, cognitive training programs targeting core EFs (inhibition, working memory, switching) and higher-order EFs (planning, reasoning) typically yield significant but small effects and limited far transfer (Bombonato et al., 2024; Scionti et al., 2020). However, there is evidence that ecological, curriculum-embedded problem-solving activities that naturally engage executive functions and expose children to meaningful challenges may be more effective (Diamond & Lee, 2011; Traverso et al., 2019).

Among curriculum-based programs, computational thinking—an approach to problem-solving modeled on computer science—has been shown to improve executive functions in typically developing primary school children (e.g., Montuori et al., 2024). Often implemented through coding games in school, computational thinking involves analyzing and decomposing problems into their constituent elements and developing step-by-step procedures (algorithms) and corresponding instructions (code) to solve them. Although studies consistently show that these activities are effective at stimulating cognitive skills in typically developing children (Montuori et al., 2024; Scherer et al., 2019), their efficacy for children with cognitive disabilities or neurodevelopmental disorders remains unclear, and research in this area is limited (Bargagna et al., 2019; Di Lieto et al., 2020a). In this paper, we present a multiple-baseline single-cases study examining the cognitive benefits of computational thinking and coding for three children with sickle cell disease, a condition typically characterized by significant executive dysfunction and early neurocognitive deficits similar to those of neurodevelopmental disorders (Schatz & McClellan, 2006). 

### 1.1. Executive Function Impairment in Children with Sickle-Cell Disease

Sickle cell disease (SCD) is a rare inherited hemoglobin disorder, prevalent in African regions, and characterized by chronic hemolytic anemia and recurrent vaso-occlusive events that may lead to cerebral hypoxia and silent or overt strokes in children (Prussien et al., 2019). In Western countries, where many affected children come from immigrant backgrounds, SCD is also associated with language difficulties typical of second-language (L2) learners (Arfé et al., 2018). Disease severity generally varies by genotype. Homozygous HbS disease (HbSS) and HbS0 thalassemia show lower hemoglobin, earlier cerebral vasculopathy, and a generally more severe clinical profile with higher cerebrovascular risk than heterozygous variants (HbSC, HbSβ) (Schatz & McClellan, 2006). Because vaso-occlusion and hypoxia often affect frontal brain regions in both HbSS and HbSC genotypes, executive function deficits are considered the primary neurocognitive sequelae of SCD (e.g., Schatz et al., 2002; Prussien et al., 2019).

Across development, children with SCD show a progressive decline in executive functioning due to the cumulative effects of overt and silent infarcts, chronic anemia, and hypoxia; consequently, school-aged children are typically more affected than preschoolers (Downes et al., 2018; Prussien et al., 2019; Reggiani et al., 2025). Common difficulties involve attention, working memory, and processing speed (Downes et al., 2018; Smith & Schatz, 2016; Stotesbury et al., 2018), with additional risks for verbal reasoning and fluency observed in school-age samples (Arfé et al., 2018; Prussien et al., 2019; Schatz et al., 2002).

These neurocognitive complications in SCD are primarily addressed through medical treatments such as hydroxyurea, which reduces vaso-occlusive crises, or hematopoietic stem cell transplantation (Sahu et al., 2022; AIEOP, 2023). However, cognitive training represents an important complementary intervention, especially for pediatric populations (Reggiani et al., 2025).

Despite guidelines recommending cognitive enhancement interventions (Hoyt et al., 2023), few cognitive programs addressed to children with SCD have been systematically tested to date (Allen et al., 2020; Hardy et al., 2021; Hurley et al., 2025), and attempts to implement such interventions have encountered difficulties with patient adherence, limiting their overall effectiveness (Hoyt et al., 2023). In their systematic review, Hoyt et al. (2023) identified only four cognitively focused behavioral interventions for children with SCD, three of which targeted memory skills. These protocols primarily relied on computerized training (Allen et al., 2020; Hardy et al., 2021). For example, studies by Hardy et al. (2016, 2021) reported promising results from a validated computerized program—Cogmed Working Memory Training—with improvements in verbal memory (*d* = 0.50), working memory (*d* = 0.70), and transfer to mathematical reasoning (*d* = 0.70) in children aged 7–16 years. However, adherence to the program was low. 

### 1.2. Computational Thinking and Coding Programs for Cognitive Training

Studies on cognitive disabilities and neurodevelopmental disorders show that executive function training can yield positive near transfer and small-to-moderate far transfer effects on clinical symptoms (effect size = 0.33, Bombonato et al., 2024). However, among cognitive training approaches, narrowly focused practice targeting specific EFs is generally less effective than educational programs that emphasize self-regulation and problem-solving through curricular activities, such as Montessori or Tools of the Mind (Diamond & Ling, 2016; Scionti et al., 2020; Takacs & Kassai, 2019). Since EF development relies on adapting to changing task and environmental demands, comprehensive educational programs that provide meaningful and varied cognitive challenges are key drivers of this development (Diamond & Lee, 2011; Diamond & Ling, 2016; Traverso et al., 2019). Computational thinking and coding programs, via virtual coding or educational robotics, are promising in this regard.

Computational thinking is defined as a problem-solving process (Wing, 2006) that involves higher order cognitive abilities, such as (1) analyzing and decomposing problems into parts, (2) planning a sequence of steps for their solution (algorithms) and generating instructions for executing the sequence (coding); and (3) identifying and correcting errors in solutions (debugging, Arfé et al., 2019; Wing, 2006). Computational thinking activities designed for schoolchildren typically involve coding, that is, planning and generating instructions to solve digital or unplugged problem-solving games ranging from highly structured to more open-ended, such as guiding a character to reach a target in a digital environment, programming a robot to achieve a goal, or producing a story (e.g., through apps such as Scratch). These cognitively engaging and challenging activities appear to be highly effective in stimulating and enhancing children’s executive functions, even when compared with traditionally effective EF training programs (Montuori et al., 2024).

Because computational thinking and coding involve problem analysis and planning, which in turn engage working memory resources and inhibitory control (Diamond, 2013), coding activities may affect both higher-order EFs (e.g., planning), and core EF skills (e.g., cognitive inhibition). This seems supported by empirical studies reporting positive effects of computational thinking interventions across executive function domains in early elementary school children (Arfé et al., 2019, 2020; Di Lieto et al., 2020b) and in preschoolers (Di Lieto et al., 2017). Reported effect sizes range from large (*d* > 1) for planning (Arfé et al., 2020), to moderate for core EFs, such as response inhibition (*ds* between 0.43 and 0.71, Arfé et al., 2020; Di Lieto et al., 2020b), and working memory (*d* = 0.63, Di Lieto et al., 2020b). Although for certain executive function measures (e.g., inhibition) the observed effects are not always consistent within and across studies (Arfé et al., 2020; Di Lieto et al., 2020b), a recent meta-analysis confirmed an overall positive influence of computational thinking on children’s executive functioning (Montuori et al., 2024).

In educational settings computational thinking can be practiced through paper-and-pencil tasks (unplugged coding), programming educational robots to perform tasks in the physical world (Bers et al., 2014; Montuori et al., 2023), or through visual block-based programming in digital environments (virtual coding) to solve game-like problems on platforms such as *code.org* (Arfé et al., 2020). Past randomized controlled trials (Arfé et al., 2019, 2020) provide empirical evidence of the effectiveness of *code.org* games for teaching computational thinking to early primary school children. These studies also offer guidance on how the platform’s resources and virtual coding activities can be used to enhance executive functions (EFs). However, the feasibility and efficacy of virtual coding for children with cognitive disabilities or neurodevelopmental disorders remain uncertain.

The few pioneering studies examining the cognitive effects of coding in this population have primarily involved educational robotics with small, heterogeneous samples of children with special needs (e.g., Bargagna et al., 2019; Di Lieto et al., 2020a). and have not examined individual cases or individual responses to the interventions in detail. Robust subject-level analyses, as used in single-case studies (see Tate et al., 2016, SCRIBE statement), can provide a stronger basis for evaluating the individual effectiveness of instructional approaches in special education (Maggin et al., 2018) and offer insights for individualized clinical interventions. The multiple-baseline single-case study presented in this paper provides an analysis of the learning trends and cognitive (EF) outcomes of three children with SCD following a coding intervention based on *code.org* games.

Coding may be a valuable intervention for these children for several reasons: (1) it involves playful problem-solving that challenges EFs while sustaining engagement (Montuori et al., 2024); (2) as part of the school curriculum, it is socially meaningful; (3) online platforms like *code.org* allow for flexible use of coding across diverse settings (school, home, or hospital), which is particularly important for children who may not attend school regularly because of hospitalization or medical conditions; and (4) coding games, particularly those offered by *code.org*, provide clear visual, in addition to verbal, feedback, making them suitable even for beginning readers and for children with language difficulties, which are common in SCD (Arfé et al., 2018).

### 1.3. Study Aim and Research Questions

The main goal of the study was to assess the feasibility of a cognitive training program based on *code.org* with three children with SCD and to derive indications for individualized intervention. The study addressed the following research questions:

1. Could a coding-based intervention be suitable for the children with sickle cell disease (SCD) involved in the study? Specifically, could the three children effectively learn through coding activities?
2. Could a short coding-based intervention be associated with improvements in the children’s verbal and nonverbal executive functions?
3. How do the children’s neuropsychological profiles modulate the effects of the intervention?

## 2. Participants and Methods

### 2.1. Participants

The participants were three children aged 7–8 years (two girls and one boy), recruited on the basis of the following inclusion criteria:

(a) a diagnosis of homozygous (HbSS) or heterozygous (HbSC) SCD
(b) age 7–8 years and enrollment in Grade 2 or 3
(c) EF impairments reported in the most recent clinical examination
(d) Italian as the dominant language
(e) family/child compliance: defined as availability to participate regularly in the baseline, assessment, and intervention phases for the full duration of the study.

Two children had the homozygous HbSS genotype and one had the heterozygous HbSC genotype. All three were born in Italy to immigrant families (Cameroon, Togo, Algeria) and were in their third year of primary school. None showed evidence of stroke-related injury or major cerebrovascular events, and no significant sleep-related problems were reported by the participants or their parents. See Table 1 for participants’ characteristics and cognitive profiles.

P. has homozygous sickle cell disease and is on regular hydroxyurea therapy. She is an only child, with a mother affected by SCD, and experienced three hospitalizations for vaso-occlusive crises (VOC) in the year prior to the coding training. At the time of the training, she was receiving school-based tutoring for language difficulties (including reading and writing), as well as home-based assistance with homework.

V. has heterozygous sickle cell disease and does not take regular medications other than pain relievers. She has had only one hospitalization for VOC since birth. Cognitive assessment, however, revealed a profile consistent with cognitive weaknesses, and the school reported difficulties in reading and comprehension. At the time of the training, she had not yet initiated a formal clinical evaluation for learning difficulties and was not receiving support for academic problems at school or at home.

N. has a homozygous genotype, with previous cerebral vasculopathy (no stroke, but persistent abnormal Transcranial Doppler Velocities-TCD) and entered the trial eight months after hematopoietic stem cell transplantation (HSCT). Owing to post-transplant pharmacological treatment and monitoring protocols, his clinical condition was unique, even in comparison with the other two participants. The post-transplant acute phase (6–10 months) involves frequent hospital visits and possible admissions. Because of immunosuppression, at the time of the trial N. attended his third year of schooling through online learning, supported by a home teacher, and was receiving corticosteroids and immunosuppressive medication. His participation in the training was planned to provide additional support for the enhancement of executive function during the post-transplant period. Although HSCT is generally curative and considerably reduces vaso-occlusive phenomena (Walters, 2015), neurocognitive dysfunctions may persist in children (Kelly et al., 2018). For N., following the acute post-transplant phase, an evaluation for suspected Attention-Deficit/Hyperactivity Disorder (ADHD) was recommended and initiated.

All children had TCD values within the normal range at the time of the intervention; however, N had previously presented an abnormal TCD, which was the reason for undergoing HSCT. As shown in Table 1, all children exhibited a cognitive profile characterized by IQ scores in the borderline range. P. demonstrated weaknesses in verbal comprehension and working memory; V. in processing speed and working memory; and N. in verbal comprehension. For all three participants, perceptual reasoning skills (PRI) emerged as a relative strength (P. = 119; V. = 82; N. = 100). Fatigue—a common symptom in sickle cell disease (SCD)—was reported by parents for all children. None of the children had prior exposure to coding, and all reported comparable levels of familiarity with technology, as assessed via questionnaire.

All parents provided written informed consent for their children to participate in the study. In addition, we requested students’ oral consent to the study before each assessment session. The study was conducted in accordance with the Declaration of Helsinki. The protocol was approved by the Ethics Committee of the Province of Padova (Project identification code 3068P) in March 2014. The informed consent was subsequently updated to ensure compliance with the GDPR.

### 2.2. Design and Procedure

This single-case study employed a standard multiple-baseline AB (baseline–intervention) design and adhered to the methodological recommendations outlined by the What Works Clearinghouse (WWC, 2022; *Version 5*) and Ganz and Ayres (2018), including: (1) a minimum of three baseline assessments initiated at different points in time for each case to demonstrate intervention effects; (2) stability in baseline trends or the use of statistical methods to correct baseline trends; (3) the integration of visual and statistical analyses; (4) robust effect-size estimation (e.g., Tau-U); and (5) the assessment of both intervention fidelity and social validity.

The AB baseline–treatment design was complemented by pre- and post-intervention neuropsychological assessments to examine whether potential effects of the coding intervention were also reflected in neuropsychological measures of executive function (EF) in the three cases.

*Baseline-treatment design.* All three children completed a coding baseline phase using *code.org* games to assess their baseline coding performance. To determine whether any observed improvements were attributable to the introduction of the intervention or to practice effects, the duration of the baseline phase varied across participants. Accordingly, the transition from the baseline to the intervention phase occurred at different times for the three participants: after three, six, or nine baseline observations (WWC, 2022). Because *code.org* games naturally provide visual and verbal feedback, the baseline phase was kept as short as possible (see also Section 2.2.1). The complete training plan, defined on the basis of prior research (Arfé et al., 2020), is described in Table 2. It consisted of 11 training sessions in which coding games from *code.org* were performed with experimenter scaffolding.

*Pre-post intervention neuropsychological assessment.* In addition to the coding baseline assessment, all children underwent pre- and post-intervention assessments of verbal and nonverbal EFs: verbal working memory (Verbal Digit Span, Wechsler, 2003); semantic and phonological fluency (BVN 5–11, Bisiacchi et al., 2005); nonverbal planning (Tower of London, Fancello et al., 2021; Elithorn Maze Test, Gugliotta et al., 2009); and cognitive inhibition (NEPSY-II, Korkman et al., 2007). Measuring improvements on neuropsychological EF tasks from pretest to post-test allowed us to assess whether potential treatment effects for the three children in the study could also generalize to verbal and nonverbal domains that are typically compromised in children with SCD (Prussien et al., 2019; Arfé et al., 2018) and associated with long-term academic performance (Morgan et al., 2019).

The data collection lasted approximately two months. Training effects were assessed by contrasting baseline coding trends with coding trends during treatment, following the standard multiple-baseline AB design (WWC, 2022). Potential generalization of treatment effects to the children’s executive functions (EFs) was examined by comparing performance on neuropsychological verbal and nonverbal EF tasks before and after the intervention, in relation to normative data.

This second analysis also served to assess the social validity of the intervention, as one criterion of social validity concerns whether observed changes are clinically significant—that is, likely to make a meaningful difference in an individual’s life (Snodgrass et al., 2018). Snodgrass et al. (2018) recommend three methods to assess social validity: (1) participants’ perceptions of intervention outcomes; (2) normative (or social) comparison, which consists of comparing the participants’ post-intervention outcomes with those of a reference group (with typical development); and (3) maintenance/sustainability measures, assessing whether the benefits from an intervention continue after the intervention is completed. We used a normative comparison approach. Given the key role of EF skills in children’s life and academic achievements (Morgan et al., 2019), significant improvement in this domain relative to normative data from typically developing children would have clear clinical significance. In addition, children’s subjective perceptions of their personal improvement in coding were monitored throughout the intervention by asking them to self-assess their performance.

The study protocol is summarized in Table 2. Pre- and post-intervention neuropsychological assessments were conducted by a trained neuropsychologist during hospital visits, while coding baseline and treatment phases were carried out at participants’ homes by the fifth author (S.C.).

#### 2.2.1. Coding Baseline and Treatment Phases

Coding activities for the baseline and treatment phases were selected from *code.org*, which introduces computational thinking through block-based problem-solving games targeting core concepts such as sequencing, loops, and debugging. Course C (2021), recommended for second graders, was used to reduce reading demands and ensure the feasibility of the training for all children.

An advantage of *code.org* is that it is among the most widely used platforms worldwide for teaching coding in schools and offers teachers structured sequences of coding lessons which are appropriate for primary school children (Montuori et al., 2024). Games are translated in more than 20 languages, which allows replication studies and the implementation of successful coding programs across countries.

**Figure 1:**
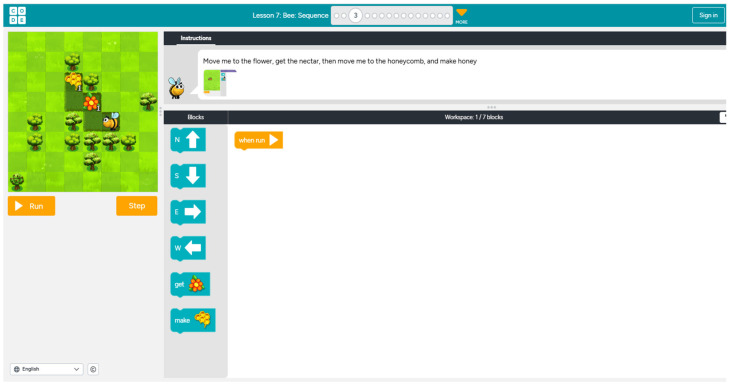
Lesson 7, course 1 (https://studio.Code.org/s/course1/stage/7/puzzle/3, accessed 27 May 2019).

*Code.org games.* Sequencing, loops, and debugging games were all used in the study. Sequencing games require building linear algorithms stacking code blocks together in a linear sequence to move, for instance, a sprite—e.g., a bee—from one side of a maze to a target—a honeycomb—to the other side (see Figure 1). Sequence-loop games add repeat blocks to allow repeating the same sequence of steps multiple times (e.g., Exercise 7, lesson 3, Course C). Debugging games provide faulty block-code sequences that children had to inspect, identify errors in, and correct (see Exercise 5, Lesson 4, Course C).

**Baseline.** The three tiers comprised baseline phases of 3 (P.), 6 (V.), and 9 (N.) observations, respectively, followed by 11 treatment sessions for each child. In the baseline phase, children performed sequencing, loops, and debugging coding games from Course C, *code.org*, without scaffolding, while scaffolding was added during training. Before the baseline, the children were familiarized with the *code.org* platform and drag-and-drop trials assisted by the examiner. The list and sequence of coding games used in the three baseline phases are available in Supplementary Materials S1.

The baseline and training outcome measure was accuracy on coding games. For each coding game, accuracy was scored as follows: 0 if the child failed or succeeded after more than two attempts, 1 if the child succeeded on the second attempt, and 2 if the child succeeded on the first attempt.

As *code.org* games are designed to support learning through adaptive visual and verbal feedback and planned hints provided when programming errors occur, the baseline phase was kept as short as possible: three data points for P., six for V., and nine for N. (see Table 2 and Supplementary Materials S1).

**Treatment/training phase.** The transition from the baseline to the intervention phases occurred at different times for the three participants (Table 2). The training phase consisted of 11 training sessions, each lasting approximately 45 min and involving 4–5 pre-selected coding games from Lessons 3 to 9 of Course C (2021), with the exception of the last three sessions, which each involved a single coding trial identical to those used in the first three baseline observations. This was also meant to assess improvement in performance on the same problem-solving games. Due to the approaching summer break (the treatment phase ended in early July), the last three training sessions were kept shorter. Treatment sessions spanned 4–5 weeks. Breaks with play activities were provided when participants showed fatigue. Moreover, as fatigue symptoms were reported by parents for all children, session duration, frequency and the number of coding games per session were planned with this factor in mind.

Based on past research (Arfé et al., 2020), the training plan was designed with a progression that balanced gradual increase in difficulty, repeated practice and variety in coding games. While varying exercises and scenarios can enhance engagement and support cognitive flexibility and skill generalization, repeated practice is necessary to consolidate programming concepts (e.g., loops) and to strengthen self-confidence and on-task motivation (see Arfé et al., 2020; Montuori et al., 2025).

Trials were chosen to ensure variability both within and between sessions, with changes in the trained coding functions (sequences, loops, debugging) and in scenarios and sprites (Angry Birds, Scrat, Laurel, see Course C, 2021). These variations supported a problem-solving approach and encouraged generalization. Game complexity gradually increased throughout the training. The full progression of coding exercises used in the training phase is provided in Supplementary Materials S1.

*Experimenter support during the training phase.* During the training phase, the fifth author (trainer) offered scaffolding and informative feedback, such as modeling the strategies suggested by *code.org* or redirecting attention to specific task elements. These interventions emphasized careful analysis of the game goal or problem and of the relevant steps in algorithmic thinking (e.g., “*What’s the goal here?*”, “*Let’s read together the problem task*”) and debugging (e.g., “*Remember: we need to examine the code block sequence, block by block, to find what is wrong*”). Apart from this scaffolding, the child was left to attempt to solve the games autonomously. Each game solution was discussed with the researcher before moving on, including consideration and comparison of alternative possible solutions. As children’s skills improved, the experimenter gradually reduced support. As with baseline accuracy, accuracy scores ranged from 0 to 2. Mean accuracy scores were computed by averaging accuracy across the trials in each session.

**Treatment Fidelity.** A rubric and checklist were used to record deviations from the lesson plan. All three children received the same treatment program and session/games sequence, with minor deviations never exceeding 10%, ensuring equivalent treatment for all participants. The only deviations consisted of shifting two training sessions, one for V. and one for N., due to hospitalization for a vaso-occlusive crisis (VOC), and missing one session due to a medical visit. The first and fifth authors met weekly to discuss issues arising during the intervention. Apart from session rescheduling, no other significant changes occurred. 

#### 2.2.2. Neuropsychological Pre- and Post-Intervention Assessment

Six neuropsychological tests were used to assess participants’ verbal and nonverbal executive functions (EFs) before and after the intervention. By assessing both verbal and nonverbal EFs, we examined how potential intervention effects in the three children could generalize across executive function domains. Post-intervention improvements in tasks requiring EFs that were directly trained during the intervention (i.e., planning) were considered suggestive of near transfer effects. In contrast, changes in secondary cognitive functions that, although involved in the training activities, were not directly targeted by the intervention (e.g., response inhibition or verbal fluency) were considered suggestive of far transfer effects (Bombonato et al., 2024). Far transfer outcomes included response inhibition, switching, verbal and nonverbal working memory, and verbal (semantic and phonological) fluency. An extended description of the neuropsychological tests used is provided in Supplementary Materials S2.

**Nonverbal EFs.**

***Tower of London (ToL) (Fancello et al., 2021).*** Performance on the ToL is considered a valid measure of problem-solving and nonverbal planning. Accuracy scores were computed, and z-scores based on normative data were used to assess clinical improvement following the intervention. Test–retest reliability is r = 0.57.

***Elithorn maze test (BVN 12–18 Batteria per la Valutazione Neuropsicologica, Gugliotta et al., 2009).*** The Elithorn assesses nonverbal planning skills. Although the BVN Elithorn task is standardized only for ages 12–18, it has been successfully used with younger children, including six- and seven-year-olds (Arfé et al., 2019, 2020). Accuracy was computed as the number of items solved in 2 min. Test–retest reliability is moderate (r = 0.46 in the BVN manual; r = 0.41 in Arfé et al., 2020), a level typical for some executive function tasks given their low stability over time (Paap & Sawi, 2016; Weafer et al., 2013). Z-scores were calculated using performance data from second graders reported in Arfé et al. (2019).

***NEPSY-II response inhibition (Korkman et al., 2007).*** The NEPSY-II Shapes and Arrows subtest was used to assess inhibition and switching. For both tasks, accuracy (total errors, including omissions and self-corrections) was recorded. The test is standardized for ages 3–16 and shows good reliability (r = 0.77 for inhibition errors). Percentile ranks were derived from normative data.

**Verbal EFs.**

***Verbal Digit Span (Wechsler, 2003).*** The digit span (forward and backward condition) assesses verbal working memory. Raw scores were converted to scaled scores using age-based WISC-IV norms. Reliability, for the WISC-IV subtest, is good (r = 0.79 for forward; r = 0.74 for backward digit span).

***Semantic fluency (BVN 5–11, Bisiacchi et al., 2005).*** The semantic fluency task assesses the efficiency and accuracy of retrieving semantically related concepts and words, a verbal process that—along with phonological fluency—entails executive control (Friesen et al., 2015) and executive function skills (Amunts et al., 2020) and is often impaired in children with sickle cell disease (Arfé et al., 2018). Children name as many items as possible within one minute for a given category (e.g., animals, foods, transportation).

***Phonological fluency (BVN 5–11, Bisiacchi et al., 2005).*** Like semantic fluency, phonological fluency measures verbal executive functioning. Children generate as many words as possible beginning with a target phoneme (e.g., *c*, *s*) within one minute. In addition to fluency and efficient retrieval, the task requires response inhibition, as children must suppress the automatic activation of semantically related words that do not meet the phonological criterion. For this reason, phonological fluency is considered a better measure of verbal EFs (Arfé et al., 2018). For both fluency tasks, the accuracy score is the number of correct words produced in 60 s. Z-scores were computed from normative data. Test–retest reliability is r = 0.83 for phonological and r = 0.85 for semantic fluency.

All standardized tasks were administered before and after the training.

### 2.3. Analytic Procedure

Response to the coding intervention was first examined by analyzing performance trends in coding across the baseline and intervention phases, and subsequently analyzing gains in nonverbal and verbal EFs.

#### 2.3.1. Baseline-Intervention Trends

In line with WWC recommendations, both visual and statistical data analyses were performed. Baseline-intervention trends were first visually scrutinized following the procedure recommended by Petersen-Brown et al. (2012) and Chen et al. (2016). The procedure involves visually assessing phases (baseline/intervention) according to four indicators: immediacy, variability, trend, and level. The immediacy criterion entails that an observable difference exists between the three last data points of Phase A (baseline) and the three first data points of Phase B (intervention). The variability criterion assumes a difference in data variability between Phase A and B. Variability is assessed relative to the mean of each phase (Phase A and B). Trend is whether there is a difference between the slope of the data in Phase A and Phase B. Level is whether there is a difference between the mean of Phase A and Phase B (Chen et al., 2016).

#### 2.3.2. Effect-Size (ESs) Computation

The second analysis step involved computing nonparametric effect sizes using a bottom-up approach (Chen et al., 2016): single nonparametric ESs for baseline–intervention contrasts were calculated for each participant to estimate individual intervention effects; then data from individual phases contrasts were combined to obtain an overall effect size for the design.

Brossart et al. (2014) recommend reporting multiple effect sizes (ES) in single-case studies. Reporting multiple effect sizes is important not only to provide a more comprehensive view of the data from comparison trials but also to ensure the robustness of the findings. In this study, two complementary ES metrics were used: IRD (improvement rate difference) and Tau-U, Tau_novlap_ (Tau-U_A vs. B_, Parker et al., 2011a, 2011b). IRD, the difference in improvement rates between baseline (Phase A) and intervention (Phase B), is particularly appropriate for discriminating intervention effects of small and moderate magnitude (Chen et al., 2016). Tau_novlap_ is the percentage of nonoverlap minus overlap (Parker et al., 2011b) and is considered the best index for bottom-up nonparametric analyses in single-case studies, even with short data series (Parker & Vannest, 2012), being relatively robust to autocorrelations and less affected by ceiling effects (Brossart et al., 2018). It is also a conservative measure to control for Type I error in AB designs (Fingerhut et al., 2021), as differently from other non-overlap indices, such as NAP or IRD, it allows researchers to account for baseline trends.

Robust IRD and Tau-U_A vs. B_ (Tau_novlap_) effect sizes were computed using the R-package for Single Case ES (v. 0.7.3; Pustejovsky et al., 2024). IRD was calculated as the improvement rate of the treatment phase minus that of the baseline phase (IRD = IRT − IRB). For each phase, IR corresponds to the number of improved data points divided by the total points in that phase. Tau-U was computed using Parker et al.’s formulae (Parker et al., 2011a, 2011b). Tau-U_A vs. B_ (Tau_novlap_) was used instead of Tau-U corrected for baseline trends, as preliminary analyses showed no significant trends and Baseline 1 (P.) had fewer than five observations (Fingerhut et al., 2021).

## 3. Results

### 3.1. Learning Trends in Coding: Baseline-Treatment Trends

**Figure 2:**
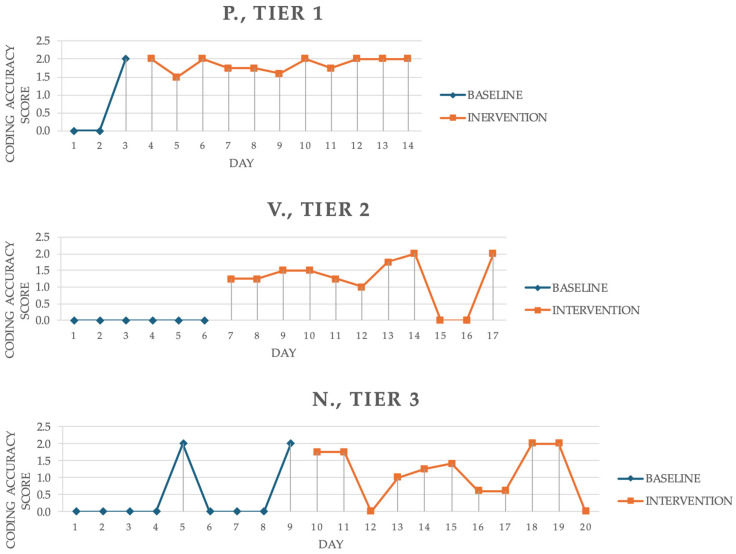
Participants’ Baseline (Blue Line) and Intervention (Red Line) Performance in Coding (P. = tier 1; V. = tier 2; N. = tier 3).

Baseline–intervention trends are shown in Figure 2. Two independent evaluators (2nd and 3rd authors) rated P. and V. trends as meeting all four criteria—immediacy, variability, trend, and level—while N. was judged to meet only trend, variability, and level. Disagreement on immediacy arose because N.’s last baseline score was at ceiling, while the third intervention score was at floor, but after discussion the evaluators agreed that N. did not meet this criterion. Interrater agreement was 83%.

A comparison of children’s performance on coding trials repeated from the baseline to the intervention phase (i.e., the first three data points in baseline and the last three in intervention) showed improved performance on two coding trials (sequences and loops games) for both P. and N., and on one coding trial (debugging game) for V. P. already showed good performance on the third coding trial at baseline.

For P., IRD = 0.79 and Tau_novlap_ = 0.52, indicated large and moderate effects, respectively (Chen et al., 2016). V. showed large effects (IRD = 0.74; Tau_novlap_ = 0.82), while N.’s IRD was 0.60 and Tau_novlap_ = 0.45, with only IRD indicating a medium effect (Figure 2). Aggregate ESs were significant: weighted IRD = 0.70, Tau_novlap_ = 0.59, Z = 4.46, *p* < .001 (Chen et al., 2016; Rakap, 2015). In synthesis, the visual and statistical analyses showed differing responses: P. and V. exhibited clear gains in coding (In both cases IRD indexes proved good effect sizes), while N.’s outcomes were less clear and consistent, as confirmed by the nonparametric effect sizes.

### 3.2. Social Validity: Generalization of Training Effects to Executive Functions

All three children perceived gains in coding, reported enjoying the activities, and felt increasingly confident in their coding abilities throughout the training. Table 3 shows children’s standardized scores or percentile ranks for verbal and nonverbal EFs at pre- and posttest. All three participants had planning difficulties at the pretest on the Tower of London and/or Elithorn Maze test. V. scored below the 5th percentile on switching, and N. scored 2 SD below age-level on semantic fluency. Digit span was within normal range for P. and V., while N. showed borderline performance at post-test.

For the NEPSY-II Inhibition and Switching subtests, accuracy scores (numbers of total errors) are reported with the relative percentile rank, whereas for all other tests, accuracy is expressed as z- or scaled scores.

**Figure 3:**
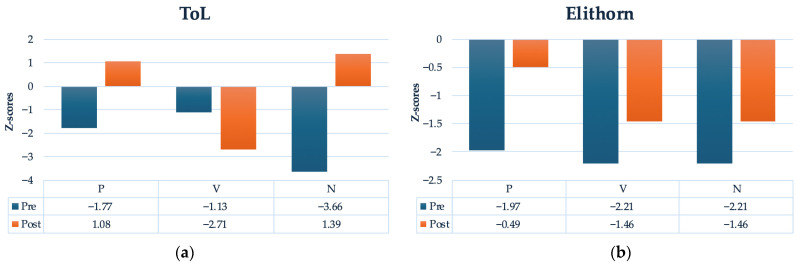
Participants’ Pretest-Posttest Improvement on: (**a**) the ToL Task and (**b**) the Elithorn Task.

After the training, all children showed some significant gains in planning, exceeding 0.50 z-scores. Notably, P. and N.’s performance on the ToL improved by 2.85 and 5.05 z-scores respectively. Only V.’s performance worsened, decreasing by 1.58 z-scores. However, on the Elithorn Maze test, all three children showed significant improvement: P. improved by 1.48 z-scores, while V. and N. each improved by 0.75 z-scores. Although children’s performance on the planning tasks was significantly and clinically below normative data at the baseline, after the training performance was within normal range (Figure 3a,b). Only V. showed improvement in switching, increasing from a score of 23 (below the 5th percentile) at baseline to a score of 4 at posttest. The other children were already performing within the normal range at pretest.

With the exception of N.’s baseline semantic fluency, children’s baseline verbal EF scores were also within the normal range. However, both N. and P. showed gains in verbal fluency. Specifically, N. improved by 1.04 z scores in semantic fluency and by 0.51 z scores in phonological fluency, whereas P. showed an improvement of 0.47 z scores in phonological fluency.

Notably, these outcomes suggest that all children—independently of their relative gains in coding—showed some significant improvement in higher-order EFs following the training. Gains were particularly evident in the areas of greatest weakness and, in some cases, were substantial, exceeding 2 z-scores.

## 4. Discussion

In this multiple-baseline single-case study, we tested the feasibility of problem-solving activities drawn from an educational coding program (*code.org*) to support executive functions in children with sickle cell disease (SCD). Preliminary evidence of intervention feasibility and efficacy was derived from observations of learning trends and improvements in EFs in three children with SCD. As anticipated earlier in this paper, studies on cognitive programs for this population remain scarce, and evidence of their effectiveness is still very limited (Allen et al., 2020; Hardy et al., 2021). Because we were interested to derive indications for individualized intervention, we conducted a single-case analysis of individual responses to an experimental coding program designed to accommodate the children’s specific needs (Maggin et al., 2018).

Although SCD involves early neurocognitive dysfunctions that resemble those observed in neurodevelopmental disorders (Schatz & McClellan, 2006), children with SCD present distinctive clinical and educational challenges compared with children with neurodevelopmental disabilities. They show progressive cognitive decline (Prussien et al., 2019), often experience executive dysfunction alongside socio-economic and linguistic disadvantage (e.g., Arfé et al., 2018), and face reduced adherence to interventions due to factors such as poor family resources (i.e., socio-economic disadvantage) and recurrent hospitalizations (Hoyt et al., 2023). Although our participants shared these risks, their cognitive profiles differed. The single-case design enabled an analysis of each child’s specific and differential response to the intervention as a function of their clinical and cognitive characteristics.

### 4.1. Response to the Coding Intervention: Feasibility, Adherence and Efficacy

All three children found the coding activities engaging and completed 100% of the sessions, adapting also to deviations from the original program due to medical visits or hospitalization. Adherence was therefore high compared with prior cognitive-training studies in pediatric SCD; for example, only 15% of participants in Hardy et al. (2021) completed all sessions recommended by the Cogmed program.

It must be noted that our program was shorter (11 vs. 20 sessions), less frequent (twice weekly vs. three to five times per week), and delivered entirely with experimenter support. In contrast, Cogmed was largely self-administered. A key difference lies in the level of scaffolding: while Cogmed coaches offer remote monitoring, they do not provide individualized support. In our study, scaffolding was integral to the coding sessions, mirroring typical classroom implementations where teachers guide and supervise students.

Two weekly sessions in our training program represented a sustainable effort even for young children, and, as will be discussed below, may have been sufficient to support some improvement in the children’s higher-order EFs. The nature of *code.org* activities, that could be easily performed online, across different settings (at home, school or in the hospital) and the collaboration between the experimenter and the hospital represented two strengths of this intervention trial.

Overall, the aggregate effect sizes indicated a positive and significant effect of the intervention, exceeding the cutoff thresholds identified by Chen et al. (2016) for both IRD and Tau-U indices. However, the response to the intervention varied across the three children. Both the visual analysis of baseline-intervention phase contrasts and the effect sizes computation confirmed clear learning trends and significant intervention effects for both P. and V., with robust improvement rate difference (IRD) values of 0.79 and 0.74, considered large, and Tau_novlap_ indices of 0.52 and 0.82, both exceeding the 0.47 cutoff threshold indicating moderate to large effects (Chen et al., 2016). In contrast, the visual trends appeared less consistent and the nonparametric effect sizes were smaller for N., with only the IRD index indicating a moderate effect (0.60).

### 4.2. Social Validity: Generalization to Executive Functions

In line with the expected profile of children with poor EF skills, all three participants showed significant planning difficulties at baseline, performing approximately two standard scores below age expectations. Notably, despite differences in coding gains, all three exhibited significant improvements in planning. That is, each participant seemed to benefit cognitively from the short coding training. Even N., who showed less consistent coding progress, demonstrated significant gains in ToL and Elithorn performance.

Considering the short duration of the intervention, gains on both planning tasks (ToL and Elithorn) were sizable, exceeding 0.50 z-scores and, crucially, reflecting a shift from performance well below the clinical cut-off (−1.5 z-scores) at baseline to performance within the normal range after training. For P. and N., improvements in planning exceeded 2 z-scores. Notably, N. showed the largest gains in planning on the ToL, a finding that could be attributed to the combined effects of HSCT and the cognitive intervention (Kelly et al., 2018). V. was the only participant to show a decline in ToL performance, with scores decreasing by 1.37 z-points from pretest to posttest. This decline may be attributable to a vaso-occlusive crisis that occurred at the end of the intervention. 

Although gains of the magnitude observed in this study over such a short period of time could be considered clinically significant (e.g., Peacock et al., 2021), other factors, such as practice effects related to the test–retest procedure or increased engagement and motivation during post-intervention testing, may have contributed to the children’s improvements. Higher-order EF tasks requiring substantial strategic control (e.g., ToL, Elithorn) rarely show comparable gains from practice alone, and the five-week interval for V. and N. likely minimized memory-related effects. Nonetheless, the lack of follow-up and the short duration of the intervention suggest caution in interpreting these findings, which merit further investigation in future studies. 

Verbal EF skills were less delayed than nonverbal skills and mostly within the normal range. Nevertheless, P. and N. improved by ~0.50 standard points in phonological fluency. Moreover, N., who had a relatively low VCI on the WISC-IV and a pretest semantic fluency delay (−2.06 z), gained 1.04 z-scores after the intervention (possible explanations for this relatively large effect are discussed below).

These outcomes suggest the possibility of generalization of the coding intervention’s effects across nonverbal and verbal domains in the participants. Verbal fluency, defined as the efficiency and accuracy with which words and concepts are retrieved, entails executive control (Friesen et al., 2015). Consistently, several studies have reported strong associations between semantic and phonological fluency and nonverbal executive functions—such as planning, response inhibition, and switching—in typically developing individuals (Amunts et al., 2020) as well as in children with sickle cell disease (Arfé et al., 2018). The potential generalization of coding effects to verbal fluency abilities that are not directly trained by coding would be consistent with these findings and could plausibly reflect indirect training effects mediated by children’s inhibition and switching (Amunts et al., 2020). By engaging higher-order executive function skills, such as problem solving and planning, coding activities may also strengthen core EF skills—particularly inhibition and switching—which in turn may support performance on verbal fluency tasks (see Amunts et al., 2020; Arfé et al., 2020; Schäfer et al., 2024).

In this study, only V. showed significant improvements in switching; however, she was also the only participant who did not exhibit transfer effects to verbal fluency tasks. Among the three children, V. showed the strongest language skills (VCI = 92), which may also have supported her performance on verbal fluency. N. and P., who showed improvements in semantic and phonological fluency and in phonological fluency, respectively, did not exhibit parallel gains in inhibition or switching skills following the coding intervention. However, this does not preclude a potential contribution of inhibition or switching processes to their improvements in verbal fluency. Training effects could have been more subtle: by engaging higher-order executive functions, such as planning, coding activities may enhance children’s ability to focus and to more effectively deploy their cognitive resources—including inhibition and cognitive flexibility—when performing both nonverbal and verbal EF tasks. This also represents a plausible explanation for the significant effects observed over a short time. Differences in executive function performance exceeding 1 z-score (e.g., in semantic fluency and planning) are unlikely to be attributable solely to practice effects. One possibility is that these gains are at least partially accounted for by improvements in children’s ability to focus. An additional avenue for future research is to examine whether improvements in nonverbal executive function (EF) skills—particularly inhibition and switching—could be differentially associated with children’s semantic and phonological fluency performance, given that these abilities may rely on partially distinct cognitive processes (Schmidt et al., 2017).

If confirmed in future studies, the possibility of enhancing verbal fluency through coding would represent a promising support for habilitation and rehabilitation programs for children with SCD. Because linguistic fluency, which is often compromised in these children (Arfé et al., 2018), is also foundational for reading and writing (Ahmed et al., 2023; Kim, 2024), improvement in this area represents an important target for clinical interventions aimed at this population.

Past research demonstrates that coding interventions can also enhance core EF components like inhibition and working memory (Montuori et al., 2024), with positive effects on inhibition also reported in children with special educational needs (Di Lieto et al., 2020a). We observed no effects on these measures, likely because participants’ baseline scores were within normal range leaving little room for improvement. Di Lieto et al. (2020a) similarly reported null effects for WM in children with special educational needs. Since deficits in inhibition and WM are common in children with SCD (Hardy et al., 2021), further research on coding interventions for these skills is needed.

### 4.3. Child × Treatment Interaction: Differential Effects by Child Profile

An analysis of how individual characteristics modulate a child’s response to an intervention provides valuable insights for the design of individualized programs. The three children had distinct cognitive and EF profiles: P. showed weaknesses in verbal comprehension and working memory at the WISC-IV, significant planning deficits (with ToL and Elithorn >1.5 SD below age norms) and low, but within normal range, phonological fluency. N. had verbal comprehension and verbal EF weaknesses, with low semantic fluency, and the poorest planning performance, consistent with suspected ADHD. His phonological fluency was in the lower range, though not significantly delayed for age. V. displayed a profile consistent with general executive function deficits, characterized by weaknesses in both processing speed and working memory indexes on the WISC-IV, as well as below-age performance on planning (Elithorn) and switching tasks, whereas her verbal skills represented a relative strength.

All children benefited from the intervention, although the improvements varied depending on the child’s cognitive characteristics. P. and V., who exhibited a typical low-EF profile associated with sickle cell disease on both the WISC-IV and EF assessments—but less severe deficits in higher-order EFs—showed strong gains in coding and near-transfer effects on planning and, for V., far-transfer effects on switching—matching their relative weaknesses.

N., with the greatest baseline planning and attentional difficulties, made less progress in coding during the intervention, suggesting a minimal planning skill may be needed to engage effectively with the coding tasks. His coding performance was also more inconsistent, reflecting his attentional and self-regulation difficulties and ADHD profile (Mammarella et al., 2022). Contextual factors, such as post-transplant isolation (i.e., not attending school during the six months following the transplant) and disruptions to daily routines due to frequent hospital visits, may also have contributed to his more variable performance. The intervention’s duration and dosage may have been suboptimal for this child. Although comparable in length to prior coding-based trials (e.g., Arfé et al., 2020), successful robotics-based programs for children with special needs are typically longer (e.g., 20 sessions; Di Lieto et al., 2020a). Despite this, N. showed significant EF gains, with the largest improvements on the ToL and Elithorn planning tasks. Notably, N., who was the only child with a significant delay in verbal (semantic) fluency, was also the only participant to show significant improvements across both verbal and nonverbal EF domains (semantic fluency and planning). A combined effect of the training and the recent hematopoietic stem cell transplantation (HSCT) may have contributed to these gains. Although verbal executive function skills were not a major difficulty for P., the training appeared to be effective in the domain in which she showed relatively weaker verbal skills: phonological fluency.

### 4.4. Limitations and Future Directions

In special education, single-case studies usefully complement experimental research by providing in-depth evidence on intervention feasibility and child × treatment interactions at the individual level, though the lack of case–control comparisons remains a limitation. This multiple-baseline study supports the feasibility and potential effectiveness of coding interventions for children with EF impairments associated with SCD and highlights how treatment effects can vary depending on the child’s specific cognitive profiles. Although this single-case design provides preliminary indications for clinical intervention, randomized controlled trials are needed to confirm and extend its findings.

The short baseline for participant P. represents a limitation of this single case study. Because the coding games used in the intervention included embedded feedback, baselines were kept as short as possible. However, three observations constitute a minimal baseline, and according to WWC (2022) standards longer baselines are generally recommended to establish baseline stability.

The duration of the intervention (slightly more than one month) may also have been insufficient to ensure durable effects. However, this could not be directly assessed because of the absence of follow-up data on maintenance, which constitutes another limitation of the study. Maintenance of intervention benefits after treatment completion is an important indicator of the social validity of an intervention (Snodgrass et al., 2018). Testing the efficacy of interventions of longer duration and assessing maintenance effects through follow-up evaluations will be a priority for future studies. Additionally, future studies should seek to extend analyses of coding intervention effects to more ecologically and socially meaningful outcome measures, such as observable changes in children’s concentration, attentional control, or self-regulation in real-life settings (e.g., home and school).

A final comment pertains to the implementation of the intervention in classrooms. Although the present study suggests that coding activities can be effective in individualized, expert-guided interventions, their implementation in classrooms or when delivered by teachers was not examined. Teacher support is central to instruction; however, limited coding expertise may affect intervention outcomes. Although *code.org* environments are designed to be accessible and sufficiently structured to support teachers’ instructional planning, and may be usable with minimal training, adapting individualized coding interventions for children with special needs to classroom contexts may remain challenging. Accordingly, the feasibility of implementing such interventions in school settings needs to be tested. Embedding coding programs in classrooms is a key goal of the European Commission’s Digital Education Action Plan 2021–2027 and aligns with broader EU educational priorities (Bocconi et al., 2022). 

For children with SCD and similar conditions, it is also important to understand how variations in program duration and dosage influence individual outcomes, and how these interventions interact with medical treatments. The broader aim is to identify the most effective individualized combination of supports for each child.

## 5. Conclusions

This study was the first attempt to explore the use of coding activities as a means of counteracting the cognitive decline associated with SCD in school-aged children. Despite being a rare disease, SCD represents the most common inherited blood disorder worldwide, with globally over 300,000 newborns affected each year by the homogenous HbSS genotype. The neurocognitive deficits typical of SCD are associated with a cascade of functional limitations that globally affect the child’s quality of life (Longoria et al., 2022).

Identifying intervention programs that can help counteract the neurocognitive decline that is normally associated with SCD is crucial for the health, education and wellbeing of these children. Unfortunately, research in this area is at its early stages, and neurocognitive intervention programs are often too demanding for families who typically have limited resources (Hardy et al., 2021). Preliminary evidence suggests that neurocognitive training programs targeting specific executive functions may be relatively effective but frequently show limited transfer effects to other EFs (Diamond & Ling, 2016), and, in SCD, low adherence (Hardy et al., 2021), probably because these programs are often difficult to integrate into children’s daily routines.

Coding represents a valuable alternative to neurocognitive trainings, as it is a meaningful and motivating literacy activity that children can engage in within their everyday educational settings, at school. An additional advantage of coding is that, by posing relatively limited demands on linguistic skills and providing immediate visual feedback, it is easily accessible even to L2 learners and children with neurodevelopmental disabilities like attentional disorders or language impairment (Di Lieto et al., 2020a). The present study has provided preliminary evidence that a short intervention focused on coding activities could be feasible and effective for at least some children with SCD, and could potentially stimulate nonverbal higher-order EFs, such as planning, and, interestingly, also verbal EF skills.

Although preliminary and restricted to individual cases, these findings are nonetheless encouraging, given that gains in higher-order and verbal EF skills can transfer to everyday self-regulation skills and adaptive functioning, including academic and learning abilities (Blair, 2017). There are several ways in which future studies can build on these preliminary findings. A promising avenue for clinical research is to examine these effects longitudinally. However, future progress in this area will first depend on testing the efficacy of coding activities in well-powered randomized controlled trials involving children with sickle cell disease.

## Figures and Tables

**Table 1:** Participant Characteristics and Cognitive Profile.

	Haemoglobin Type	Age (Months)	Background	IQ	VCI	PRI	PSI	WMI
P. (girl)	HbSS	89	Cameroon	81	58	119	94	73
V. (girl)	HbSC	91	Togo	71	92	82	62	70
N. (boy)	HbSS	99	Algeria	82	68	100	91	91

Note. HbSS = homozygous; HbSC =heterozygous. Scaled scores are reported for the WISC-IV: IQ = Intelligence Quotient; VCI = Verbal Comprehension Index; PRI = Perceptual Reasoning Index; PSI = Processing Speed Index; and WMI = Working Memory Index.

**Table 2:** Single Case Study Protocol.

Coding Baseline Code.org Games Course C, 2021	Coding Training Program Code.org Games Course C, 2021	Pre/Post-Intervention Neuropsychological Assessment
**Coding Baseline** of different lengths:	**Coding Training sessions** transition to training occurs at different time points across the three tiers:	**Neuropsychological** **assessment**
P.’s baseline = 3 data points V.’s baseline = 6 data points N.’s baseline = 9 data points	P. = training starts at session 4 V. = training starts at session 7 N. = training starts at session 10	**Nonverbal EFs** Planning: ToL, Elithorn Inhibition: NEPSY-II Switching: NEPSY-II **Verbal EFs** Verbal WM: WISC-IV digit span Verbal fluency: Semantic fluency (BVN 5–11); Phonological fluency (BVN 5–11)
	Duration/L (overall length): 5 weeks Duration/N (number of coding sessions): 11 coding sessions Dose/L (session length): ~45 min Dose/N (number of coding trials per session): 4–5 coding games each session/1 coding trail last three sessions Frequency: 2 weekly training sessions	

**Table 3:** Changes in executive function (EF) scores from pre- to post-intervention (in bold: performances below −1.5 z-scores or below the 10th percentile).

Participant	Phase	ToL (z-sc)	Elithorn (z-sc)	NEPSY-II Inhib. (Errors Percentile)	NEPSY-II Switch. (Errors Percentile)	Sem. Flu. (z-sc)	Phon. Flu. (z-sc)	WISC-IV Digit Span (Scaled sc)
**P.**	pre	**−1.77**	**−1.97**	2 [51–75]	4 [>75]	0.33	−1.34	9
	post	1.08	−0.49	0 [>75]	0 [>75]	0.06	−0.87 #	13
**V.**	pre	−1.13	**−2.21**	2 [51–75]	**23 [2–5]**	−0.6	−0.98	8
	post	**−2.71**	−1.46	1 [>75]	4 [>75]	−0.2	−0.75	6
**N.**	pre	**−3.66**	**−2.21**	4 [26–50]	12 [26–50]	**−2.06**	−1.38	9
	post	1.39	−1.46	6 [11–25]	15 [11–25]	−1.02	−0.87	**4**

Note. z-sc = z-score; scaled sc = scaled scores; inhib. = response inhibition; switch. = switching; Sem. Flu. = Semantic Fluency; Phon. Flu. = Phonological Fluency; # = improvement of 0.47 z-scores.

## Data Availability

The data presented in this study are available on request from the corresponding author.

## Supplementary Materials

The following supporting information can be downloaded at: https://www.mdpi.com/article/10.3390/jintelligence14040055/s1, S1: Training protocol; S2: Neuropsychological pre- and post-intervention assessment.

## Abbreviations

The following abbreviations are used in this manuscript:

SCD	Sickle Cell Disease
EF	Executive Function
AIEOP	Associazione Italiana di Ematologia e Oncologia Pediatrica
