# Supplementary material for: Using Coding to Improve Executive Functioning in Children with Sickle Cell Disease: A Multiple-Baseline Single-Case Study
Source: J Intell. 2026 Apr 1;14(4):55. doi: 10.3390/jintelligence14040055 (PMC13117863; doi:10.3390/jintelligence14040055)
Supplement: Supplementary file 1 [file jintelligence-14-00055-s001.zip › Supplementary Materials S2.pdf]

**Supplementary Materials S2:**
**Neuropsychological Pre- and Post-Intervention Assessment (extended version)**

**Nonverbal EFs.**
***Tower of London (ToL) (Fancello et al., 2021).*** Performance on the ToL is considered a valid measure of problem-solving and nonverbal planning. The task requires to move three differently sized and colored beads across three pegs to replicate a target configuration with the fewest possible moves. Planning accuracy was scored. Accuracy is scored as 3 if the task is completed on the first attempt, 2 on the second, and 1 on the third. *Z*-scores, based on normative data, were computed to assess clinical improvement following the intervention. Test-retest reliability is $r = 0.57$ for accuracy scores and $r = 0.71$ for planning times. Concurrent validity with the Elithorn maze task, computed by Arfé et al. (2020) in a sample of about 180 children aged 5–6 years, was $r = 0.35$ —a moderate but significant correlation— suggesting that the two tests assess somewhat different aspects of nonverbal planning in children.
***Elithorn maze test (BVN 12-18 Batteria per la Valutazione Neuropsicologica, Gugliotta et al., 2009).*** The Elithorn assesses nonverbal planning skills. Although currently standardized only for adolescents aged 12 to 18, the test has also been successfully used with younger children (Leuzzi et al., 2004), including children with SCD (Arfé et al., 2018). The child is asked to connect a set number of black dots on a grid by drawing lines that follow three rules: move from bottom to top, avoid crossing grid lines, and do not retrace steps. Accuracy is computed as the number of items solved in 2 minutes. Test-retest reliability, as reported by the BVN manual, for older subjects, and computed in a recent study on a sample of 94 six-year-old children (Arfé et al., 2020), is moderate: $r = 0.46$ and $r = 0.41$, respectively. This level of reliability is not atypical for executive function tasks that depend on cognitive control—a skill often less stable over time than other cognitive abilities (Paap & Sawi, 2016; Weafer et al., 2013). Since age-appropriate normative scores were unavailable, *z*-scores were calculated based on performance data reported by Arfé et al. (2019) from second graders (M age = 6.89 years, 83 months; M performance = 9.79, SD = 4.91).
***NEPSY-II response inhibition (Korkman et al., 2007)*.** The A4 subtest (shapes and arrows) of the NEPSY-II was used to assess cognitive inhibition. The task consists of three conditions:
-naming: to name shapes (e.g., circle/square) or (up/down) arrows arranged in five rows as quickly as possible. The task provides a baseline measure of processing speed.
-inhibition: to inhibit the automatic naming response (e.g., saying "circle" for a circle), producing instead the opposite shape name (e.g., "square" for a circle).
-switching: to alternate between naming and inhibition based on predefined rules. This condition provides a measure of cognitive flexibility.
For the inhibition and switching tests, time (seconds) and accuracy (number of total errors, including omissions and self-corrections) are computed.

The NEPSY-II Inhibition test is standardized for children aged 3 to 16 years. The manual reports good reliability coefficients: $r = .79$ for inhibition time and $r = .77$ for inhibition errors.

**Verbal EFs.**

***Verbal Digit Span (Wechsler, 2003).*** The test assesses verbal working memory, asking the child to repeat digit sequences in the same order (digit forward condition), engaging short-term memory and attention, and in reverse order (digit backward condition), requiring greater executive control. Raw scores for the Digit Span were calculated by assigning 1 point for each correctly recalled sequence (forward and backward). Raw scores were converted into scaled scores using age-based normative tables provided in the WISC-IV manual. The WISC-IV subtest shows good reliability, with $r = .79$ for the forward span and $r = .74$ for the backward span.

***Semantic fluency (BVN 5-11, Bisiacchi et al., 2005)***. The semantic fluency task assesses fluency in concepts and words retrieval. Along with phonological fluency, semantic fluency is among the verbal processes impaired in children with SCD (Arfé et al., 2018). In this task, children name as many items as possible from a given semantic category (e.g., animals, foods, modes of transportation) within one minute.

***Phonological fluency (BVN 5-11, Bisiacchi et al., 2005).*** Similar to semantic fluency, the phonological fluency task measures verbal executive functioning. Children are asked to produce as many words as possible beginning with a given phoneme (e.g., *c, s*) within one minute. In addition to fluency and efficient retrieval, the task requires response inhibition, as children must suppress the automatic activation of semantically related words that do not meet the phonological criterion. For this reason, phonological fluency is considered a better measure of verbal EFs (Arfé et al., 2018). In both tasks scoring is the number of correctly retrieved words within 60 seconds. For both verbal fluency tasks, z-scores, based on normative data, were computed. Test-retest reliability, as reported by the manual, is $r =.83$ for phonological fluency and $r =.85$ for semantic fluency.

All standardized tasks were performed again at the end of the training, after one week from the end of the intervention.
