# Supplementary material for: Using Coding to Improve Executive Functioning in Children with Sickle Cell Disease: A Multiple-Baseline Single-Case Study
Source: J Intell. 2026 Apr 1;14(4):55. doi: 10.3390/jintelligence14040055 (PMC13117863; doi:10.3390/jintelligence14040055)
Supplement: Supplementary file 1 [file jintelligence-14-00055-s001.zip › Supplementary Materials S1.pdf]

Supplementary Materials S1:
Training Protocol

Coding Baseline Phase (code.org, Course C – 2021)

Session/Day	Tier 1 – P. Baseline 1	Tier 2 – V. Baseline 2	Tier 3 – N. Baseline 3
Session 1	Lesson 3 – game 9 (Sequencing)	Lesson 3 – game 9 (Sequencing)	Lesson 3 – game 9 (Sequencing)
Session 2	Lesson 5 – game 10 (Sequencing-Loops)	Lesson 5 – game 10 (Sequencing-Loops)	Lesson 5 – game 10 (Sequencing-Loops)
Session 3	Lesson 4 – game 9 (Debugging)	Lesson 4 – game 9 (Debugging)	Lesson 4 – game 9 (Debugging)
Session 4		Lesson 3 – game 11 (Sequencing)	Lesson 3 – game 11 (Sequencing)
Session 5		Lesson 5 – game 11 (Sequencing-Loops)	Lesson 5 – game 11 (Sequencing-Loops)
Session 6		Lesson 5 – game 8 (Debugging)	Lesson 5 – game 8 (Debugging)
Session 7			Lesson 8 – game 9 (Sequencing)
Session 8			Lesson 5 – game 13 (Loops)
Session 9			Lesson 8 – game 13 (Debugging)

Training sessions

Coding Training Phase (code.org, [Course C – 2021])

Same sessions and games for all participants.

Note: Games in (parentheses) are optional

**Training Session**	**Code.org games from Course C – 2021** (colours identify game type)	**Functions trained** (colours identify game type)
Session1	Lesson 3 – games 2, 5, 6, 10	Sequencing<br>Debuggining
Session 2	Lesson 3 – games 3, 7<br>Lesson 4 –games 2, 3, (4)	Sequencing<br>Debugging<br>Loops
Session 3	Lesson 4 – games 5, 6, 7, 8	Debugging<br>Loops/Debugging
Session 4	Lesson 5 – games 3, 4, 5, 7	Sequencing<br>Debugging
Session 5	Lesson 8: games 2, 5, 6<br>Lezione 9: games 4, (3)	Sequencing/Loops<br>Loops
Session 6	Lesson 5: games 6, 9, (2)<br>Lesson 8: games 7	Sequencing<br>Debugging<br>Loops
Session 7	Lesson 8: game 12<br>Lesson 9: games 5, 13	Sequencing/Loops<br>Debugging
Session 8	Lesson 9: games 6, 11<br>Lesson 4: games 7, (9)	Sequencing/Loops<br>Debugging
Session 9	Lesson 3 – game 9	Sequencing
Session 10	Lesson 5 – game 10	Loops
Session 11	Lesson 4 – game 9	Debugging
